# OOP-ESEEM Spectroscopy: Accuracies of Distances of Spin-Correlated Radical Pairs in Biomolecules

**DOI:** 10.3389/fmolb.2022.890826

**Published:** 2022-06-23

**Authors:** Tarek Al Said, Stefan Weber, Erik Schleicher

**Affiliations:** Institute of Physical Chemistry, University of Freiburg, Freiburg, Germany

**Keywords:** EPR spectroscopy, coupled radical pair, distance determination, dipolar coupling, OOP-ESEEM

## Abstract

In addition to the commonly used electron–electron double resonance (ELDOR) technique, there are several other electron paramagnetic resonance (EPR) methods by which structure information can be obtained by exploiting the dipolar coupling between two radicals based on its characteristic *r* ^−3^ dependence. In this contribution, we explore the potential of out-of-phase-electron-spin echo envelope modulation (OOP-ESEEM) spectroscopy to collect accurate distance information in photo-sensitive (bio) molecules. Although the method has already been applied to spin-correlated radical pairs in several classes of light-active proteins, the accuracy of the information obtained has not yet been extensively evaluated. To do this in a system-independent fashion, OOP-ESEEM time traces simulated with different values of the dipolar and exchange couplings were generated and analyzed in a best-possible way. Excellent agreement between calculated and numerically fitted values over a wide range of distances (between 15 and 45 Å) was obtained. Furthermore, the limitations of the method and the dependence on various experimental parameters could be evaluated.

## Introduction

One of the most fundamental goals of protein studies is to obtain a mechanistic understanding of biological function at a molecular level. Data on protein structure, the dynamics of structure elements and reaction kinetics are typically collected for this purpose. During the last decades, robust technological developments in the fields of X-ray crystallography, NMR, and cryogenic electron microscopy (cryo-EM) have contributed to the wealth of solved protein structures in the RCSB Protein Data Bank. X-ray crystallography remains the standard for obtaining crystal structure data at atomic resolution, although conclusions on the protein dynamics on various time scales in the protein’s physiological state may not directly be drawn. Cryo-EM, with its advantage of simpler sample preparation, is the currently most evolving method (Fernandez-Leiro and Scheres, 2016). Although this technique provides snapshots of a protein, dynamic properties that govern the protein’s functional output are still lacking. Solution NMR is potentially the best tool to study protein dynamics and structure; however, the technique is limited to smaller proteins or protein complexes and demands expensive stable-isotope-labeling strategies. Thus, biophysical methods are desired which provide structure data on the nanometer scale and dynamic information of biomolecules without extensive sample manipulation and preparation. Fluorescence-based methods, such as fluorescence resonance energy transfer (FRET), have proven quite powerful in this respect as they are sensitive down to the single-molecule level and provide real-time dynamics over several time scales (Loura and Prieto, 2011). However, FRET lacks precision and applicability when it comes to quantifying distances or distance changes. Therefore, other spectroscopic methods such as EPR are applied to obtain structure (and also dynamic) information on biomolecules.

The most commonly used EPR method for determining distances from dipolar couplings between radicals in biomolecules is pulsed electron–electron double-resonance (abbreviated PELDOR or DEER) (Pannier et al., 2000; Schiemann and Prisner, 2007; Jeschke, 2012), and to a lesser extent, relaxation-induced dipolar modulation enhancement (RIDME) spectroscopy (Kulik et al., 2001), or the more recently devised laser-DEER or laser-IMD techniques (Hintze et al., 2016; Dal Farra et al., 2019). By these methods, the distance between magnetic momenta is measured, which are either intrinsically present in the (bio)molecule of interest or introduced *via* selective spin labeling (Klare, 2013). Since several excellent reviews on the accuracy of the PELDOR method are available [for example, (Edwards and Stoll, 2016, Edwards and Stoll, 2018; Hustedt et al., 2018)], the focus of this study is on the applicability and accuracy of a further pulsed EPR method for distance determination: pulsed out-of-phase electron-spin-echo envelope modulation (OOP-ESEEM) EPR (Salikhov et al., 1992).

### Distance Determination in Correlated Radical Pairs

If two (or more) unpaired electron spins are present in a system, their interactions are described by the spin Hamiltonian which comprises contributions from exchange interaction, zero-field splitting, and dipolar coupling. If light-induced electron transfer takes place between a donor and an acceptor moiety of the system, a spin-correlated coupled radical pair is generated, which may be observed provided the EPR detection is sufficiently fast (Weber, 2017). Such a spin pair may be singlet (spin quantum number *S* = 0) or triplet (*S* = 1) configured depending on the multiplicity of the precursor state (see below). It should be noted in this context that although a photogenerated triplet state is also paramagnetic and may be considered as a very strongly coupled pair of spins within a molecular moiety, electron transfer is not needed for its generation.

In case the spins are strongly coupled, that is, they are within a very short distance with respect to each other, the exchange interaction describing the energy difference between the singlet state and the triplet manifold becomes very large. Hence, the triplet sublevels may be treated separately from the singlet level for the purpose of detection of EPR resonances. As a result, the zero-field splitting becomes dominant, which can be described by the two so-called zero-field splitting parameters, *D* and *E*, for a review see, e.g., Richert et al. (2017). These can be extracted from an EPR spectrum by spectral simulations. *D* and *E* depend on the average distance of the unpaired electrons, the delocalization of the wave function across the molecule, and the rhombicity of the interaction (Richert et al., 2017; Weber, 2017). However, because of the strong coupling, only a distance estimation becomes possible for pure triplets.

In case the two electron spins are more distant from each other, the exchange interaction and zero-field splitting are much weaker; one may speak of a spin pair or a radical pair. If radical pairs are formed by fast light-induced electron transfer, in which at least one electronically excited moiety acts as an electron acceptor or donor, a charge-separated state with two unpaired correlated electron spins is formed because the precursor spin multiplicity is transferred to the charge-separated state due to the conservation of spin-angular momentum. In this context, the distinction between a spin-correlated radical pair and an (uncorrelated) biradical needs to be made. Spin-correlation of radical pairs manifests itself in EPR spectra as electron-spin polarization that affects the intensities of resonant transitions. In contrast to spectra of thermally equilibrated spin states, spin-correlated radical pairs exhibit enhanced absorptive and emissive resonances provided the detection system is fast enough (Weber, 2017). This is because spin correlation has a limited lifetime. Its decay may be relatively slow as long as the distance and the relative orientation of the radical pair partners remain static. This is the case, for example, in some radical pairs formed between cofactors and/or amino acid residues within a protein framework, in polymer compositions consisting of donor and acceptor materials, or in synthetically prepared molecules with donor and acceptor moieties. Spin-correlated coupled radical pairs generated by light-induced electron transfer have thus been observed in photosystems involved in photosynthesis (Harvey and Wasielewski, 2021), in cryptochromes and photolyases (Biskup et al., 2009; Schleicher and Weber, 2012), in phototropins (Kay et al., 2003), in light-active decarboxylases (Sorigué et al., 2017), in biomimetic systems of artificial photosynthesis (Hou et al., 2019), and in polymer-fullerene blends devised for organic photovoltaics (Niklas and Poluektov, 2017).

With increasing distance *r*, the exchange interaction falls off exponentially—more strongly than the dipolar interaction, which follows an *r* ^−3^ dependence. The exchange interaction accounts for the quantum-mechanic indistinguishability of the two electrons and thus depends on the overlap of the respective orbitals. The dipolar interaction depend not only on the distance but also on the orientation of the radical pair with respect to the direction of the external magnetic field. Electron-spin polarization in spin-correlated coupled radical pairs may be understood with the coupled correlated radical pair (CCRP) formalism (Adrian, 1972; Closs et al., 1987; Hore et al., 1987). Such resonances can be directly detected using transient EPR spectroscopy (Weissman, 1982; Weber, 2017) and pulsed EPR methods such as OOP-ESEEM spectroscopy (Salikhov et al., 1992; Tang et al., 1994). All methods have their strengths and weaknesses in the determination of the relevant interaction parameters. Transient EPR spectroscopy is not ideal for extracting distance information as the dipolar interaction between the two radicals has little influence on the spectral shape but affects the amplitude of a radical-pair signature in cases of weaker couplings (Stehlik et al., 1989). OOP-ESEEM, on the other hand, is a method that is ideal for measuring distances between radicals; the basics of this technique will be summarized in brief in the following section.

### Basics of the OOP-ESEEM Method

**FIGURE 1:**
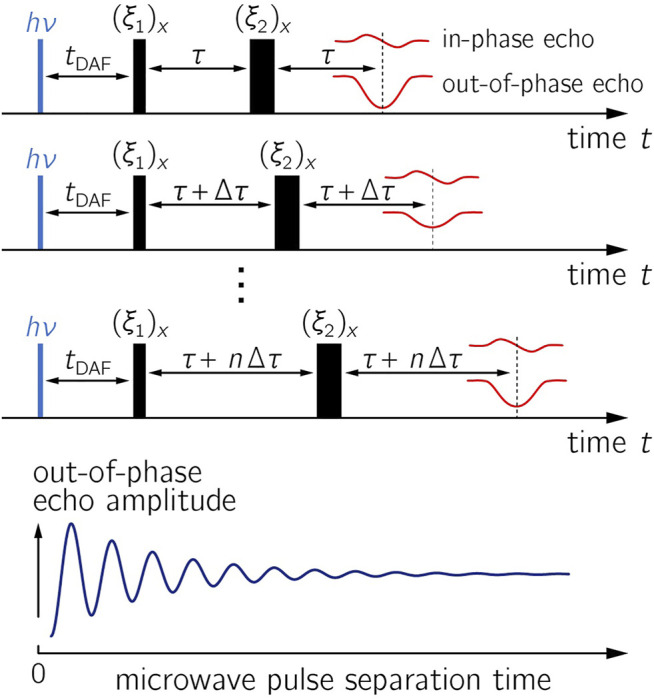
OOP-ESEEM pulse sequence. In the upper panels, the evolution of the in-phase and out-of-phase (OOP) echo components are depicted; the resulting time trace with respect to the pulse separation time is shown in the lower panel. Parameters: *t*_DAF_, time delay after the laser flash (*hν*); (*ξ*_*i*_)_*x*_, flip angles of microwave pulses with phase *x*; in-phase and out-of-phase (OOP) signals denote the signals in the *y*- and *x*-channels during quadrature detection. During the experiment, the time between the microwave pulses is varied stepwise while *t*_DAF_ is kept constant. The amplitude of the OOP signal is recorded as a function of the microwave pulse separation time.

The pulsed OOP-ESEEM EPR experiment (Salikhov et al., 1992; Tang et al., 1997) is capable of directly measuring the strengths of exchange and dipolar interactions in short-lived radical pair states. OOP-ESEEM has been previously applied to examine spin-correlated radical pairs in a number of proteins (see Table 1), composite materials designed for organic photovoltaic devices (Lukina et al., 2015), and labeled DNA hairpins (Olshansky et al., 2019). In brief, this low-temperature EPR experiment comprises a canonical two-microwave-pulse echo sequence [*hν*–*t*_DAF_–(*ξ*_1_)_*x*_–*τ*–(*ξ*_2_)_*x*_–*τ*–echo] applied after a short laser pulse that initiates radical pair formation (Figure 1). In general, the precise flip angles *ξ*_*i*_ of the pulses, which can differ significantly from 90° (and 180°) (Tang et al., 1997; Krzystyniak, 2003), should be optimized during the experimental setup. The maximum OOP-ESEEM signal can be determined by adjusting the pulse length at constant microwave power or vice versa. The echo intensity in the out-of-phase channel detected as a function of the separation time between the microwave pulses thus becomes modulated by

$$\omega_{\mathrm{mod}}=2J-2D\left(3\cos^{2}\theta -1\right)/3$$ (1)

as a result of dipolar and exchange interactions, with *θ* being the orientation of the dipolar axis with respect to the direction of the external magnetic field (Salikhov et al., 1992), and D and J are the dipolar and exchange interaction, respectively, both in angular frequency units. This modulation can be converted by sine Fourier transformation (SFT) into a frequency spectrum. From the resulting Pake doublets the dipolar and exchange couplings can be extracted at high precision from turning and inflection points of spectral simulations (Hoff et al., 1998; Fursman and Hore, 1999; Santabarbara et al., 2006). They correspond to the frequencies at which the dipolar axis is parallel and perpendicular to the magnetic field, respectively. These frequencies are given by

$$\nu_{⊥}=\pm \left(2D/3+2J\right)\;\mathrm{and}\;{\text{ν}}_{∥}=\pm \left(-4D/3+2J\right)$$ (2)

**TABLE 1:** Radical pair distances in various protein systems determined using OOP-ESSEM and comparison to the respective distances obtained from crystal structures or structure models.

Protein and Organism	Radical Pair	Distance from OOP-ESEEM/Å	Distance obtained by crystal structure (a) or by calculation (b)/Å	Reference
*Rhodobacter sphaeroides* R26 RC	[P865^•+^ Q_A_^•–^]	28.4	28.3 (a)	Zech et al. (1996); Bittl and Zech (1997)
*Synechococcus elongatus* PS I	[P700^•+^ A_1_^•–^]	25.4		
Spinach PS I	[P700^•+^ A_1_^•–^]	25.3 ± 0.3		Dzuba et al. (1997)
*C. reinhardtii* PS I	[P700^•+^ A_1_^•–^]	25.4 ± 0.03	24.5 (a)	Santabarbara et al. (2006)
Spinach PS I	[P700^•+^ A_1_^•–^]	25.4 ± 0.03	26.0 (a)	
*Synechocystis* sp*.* PS I	[P700^•+^ A_1_^•–^]	25.5 ± 0.02	24.5 (a)	
*Synechocystis* sp. PS I	[P700^•+^ A_1_^•–^]	26.1 ± 0.2	24.5 (a)	Savitsky et al. (2013)
Artificial photosynthetic systems	[BDX^•+^ PI^•–^]	25.0 ± 0.1	25.4 (b)	Carmieli et al. (2009)
	[TTF^•+^ PI^•–^]	28.1 ± 0.1	27.3 (b)	
	[DMJ^•+^ NI^•–^]	38.0 ± 0.2	37.6 (b)	
Spinach PS II	[P680^•+^ Q_A_^•–^]	27.4 ± 0.3	27 (a)	Hara et al. (1997)
	[Y_z_^•+^ Q_A_^•–^]	34 ± 1	34 (a)	Zech et al. (1997)
*Drosophila* cryptochrome	[FAD^•–^ TrpD^•+^]	22.4 ± 0.5	21.6 (a)	Nohr et al. (2017)
*E. coli* photolyase	[FAD^•–^ TrpC^•+^]	19.5 ± 0.5	20.5 (a)	
Pigeon cryptochrome 4	[FAD^•–^ TrpD^•+^]	21.2 ± 0.2	20.9 (a)	Hochstoeger et al. (2020)
	[FAD^•–^ TrpC^•+^]	17.5 ± 0.1	17.5 (a)	
European robin cryptochrome	[FAD^•–^ TrpD^•+^]	21	21.3 (a)	Xu et al. (2021)
	[FAD^•–^ TrpC^•+^]	18	17.6 (a)	

Both parameters, D and J , can hence be extracted from the two frequencies. By using the point-dipole approximation (PDA), the distance r between the radicals can be calculated based on the relation (Hoff et al., 1998; Santabarbara et al., 2005)

$$D/\mathrm{MHz}={-78.08\times 10^{3}/\left(r/\text{Å}\right)}^{3}\mathrm{or}D/\mathrm{mT}=-2786/{\left(r/\text{Å}\right)}^{3}$$ (3)

Please note that there is a factor of 3/2 between Eq. 3 and the relation commonly used in PELDOR spectroscopy to extract distance information: $\nu_{⊥}=-52.16\times 10^{3}\text{M}\text{H}\text{z}/{\left(r/\text{Å}\right)}^{3}$ (Jeschke, 2012). The reason for this is that PELDOR is typically applied to systems containing spins at larger distances ( >20Å ); hence, J is assumed zero and only one of the two frequencies ( $\nu_{⊥}$ ) of the Pake pattern needs to be read out (Reginsson and Schiemann, 2011). In this case Eq. 2 simplifies to $\left|D\right|=3\nu_{⊥}/2$ .

In comparison to other methods for distance determination, such as PELDOR spectroscopy (Jeschke, 2012), OOP-ESEEM is conceptually more straightforward and requires only two pulses of the same microwave frequency (instead of four or more pulses of different frequencies) (see below). Typically, the method is applied to the examination of short-lived spin pairs generated in an unmodified protein system: hence, any potentially structure-disturbing effects, as they may occur upon site-directed spin labeling, are avoided.

The anisotropies of the Zeeman interaction and the orientation of the two radicals with respect to each other have only a minute influence on the out-of-phase echo modulation (Bittl and Zech, 2001) as long as the experiment is carried out at not too high microwave frequencies and corresponding magnetic field strengths so that orientation-selection effects (Harmer, 2016; Schnegg, 2017) can be neglected. Furthermore, hyperfine couplings and their anisotropies have only a minor effect on the shape of the OOP-ESEEM time traces despite the fact that other ESEEM techniques are particularly sensitive to these (van Doorslaer, 2017). Theoretical studies treating the case of broadband microwave excitation in OOP-ESEEM corroborate this finding (Dzuba, 1997; Timmel et al., 1998): Anisotropic hyperfine couplings contribute only as a second-order effect to the echo modulation (Tang et al., 1995). In only a few experimental studies using the OOP-ESEEM technique slight distortions of the earliest echo modulations by hyperfine couplings were observed, although the obtained distances remained unaffected (Fursman and Hore, 1999; Nohr et al., 2017).

Information on the orientation of the dipolar coupling tensor have been previously obtained by examination of single crystals that have been rotated with respect to the direction of the external magnetic field (Bittl et al., 1997). It is conceivable that such information may also be obtained using orientation selection effects at high magnetic fields (Savitsky et al., 2007; Savitsky et al., 2013; Harmer, 2016; Schnegg, 2017), oriented samples (Hasegawa et al., 2017), or by exploiting selective optical sample excitation using polarized light (Hamada et al., 2021). However, to the best of our knowledge, such techniques have not yet been applied to OOP-ESEEM.

Theory predicts that not only singlet-configured radical pairs but also triplet-configured ones exhibit an OOP ESEEM effect. The echo amplitude of the latter is expected to assume opposite polarity and its intensity to be reduced by a factor of three (Tang et al., 1997). Thus, the sign of the echo modulation could, in principle, be used to assign the precursor multiplicity, singlet or triplet, of a given radical pair. A comparison of experimental OOP-ESEEM data from ^1^[P_865_^•+^ Q_A_^•–^] and ^3^[P_865_^•+^ Q_A_^•–^], however, revealed almost identical time traces and no indication of any polarity change (Borovykh et al., 2002; Kulik et al., 2003).

In principle, the OOP-ESEEM method benefits from the initial spin-correlation in a radical-pair generation that manifests itself in strongly polarized resonances, thereby significantly increasing the signal-to-noise ratio (SNR) of detected spin echoes and consequently of their modulation. Also, information on the precursor spin multiplicity is obtained from such experiments, thereby revealing traits of the photochemistry of a system. The method thus complements the PELDOR technique, which is widely applied to measure distances and their distributions in uncorrelated but coupled biradicals. The OOP-ESEEM experiment requires only two pulses at one microwave frequency, but a laser pulse, which is required for the generation of the radical pair by photoexcitation, needs to be applied and the microwave pulse sequence tied to it. With modern EPR instrumentation, this is nowadays straightforward. Photogeneration of radical pairs could be a problem for the measurement of distances using OOP-ESSEM if a sample suffers from photodecomposition. In such a case, only a limited SNR can be reached, and the sample needs to be renewed for increasing the SNR if available in larger quantities. Here, PELDOR has a clear advantage as the experiment can be repeated without sample loss until the desired SNR is reached. The fact that typically optical sample excitation is used for the generation of spin-correlated radial pairs (although other methods for generating a spin-correlated state are conceivable) is, however, limiting the application of the OOP-ESEEM method. Intrinsically, very few proteins are eligible as long as the addition of light-active labels and/or redox-active amino acids by biochemical and molecular biology methods is still a conceptual challenge.

## Materials and Methods

We will evaluate the applicability of OOP-ESEEM in detail by comparing distances derived from this method with those obtained using other methods for structure determination, such as X-ray crystallography. In addition, the accuracy of the method will be evaluated by analyzing artificially generated echo decay curves. Specifically, it shall be clarified how accurate the information obtained by this method is, on which length scales the method is applicable and which limitations are involved.

### Calculation of OOP-ESEEM Spectra

Our calculations of OOP-ESEEM echo decay curves were performed under the assumption of weak dipolar coupling between the radicals and the validity of the point-dipole approximation (a dipolar coupling tensor of axial symmetry). We further assumed microwave pulses that are able to excite the entire range of spectral resonances. In addition, the following simplifications were made: Both parts of the radical pair have an isotopic *g*-value of 2.0023. Hyperfine couplings were disregarded; hence, it was assumed that the calculated echo trace from the OOP channel was unaffected by additional nuclear ESEEM. Furthermore, effects of zero-quantum coherence were ignored, which can be achieved experimentally by applying the microwave pulse sequence after a sufficiently long *t*_DAF_ (see Figure 1) (Hoff et al., 1998). The *τ*-dependent echo intensity can then be determined for a disordered ensemble by

$$S\left(\tau \right)=A\;\exp\left(-\tau /T_{\text{d}}\right)\int_{0}^{2\pi }\int_{0}^{\pi }\sin\left(\omega_{\mathrm{mod}}\left(\theta \right)\tau \right)\sin\left(\theta \right)\;\text{d}\theta \;\text{d}\phi$$ (4)

Here $\omega_{\mathrm{mod}}\left(\theta \right)$ is the observed frequency modulation (see Eq. 1), θ and ϕ are the angles between the external magnetic field and the axis connecting the two magnetic point dipoles, A is the amplitude of the signal, and $T_{\text{d}}$ is the relaxation time (Salikhov et al., 1992). This integral can be rewritten as the sum of Fresnel integrals

$$S\left(\tau \right)=\frac{2\pi^{1.5}}{\sqrt{D\tau }}H\;\text{exp}\left(\frac{-\tau }{T_{\text{d}}}\right)\left[\sin\left(\frac{2\left(D+3J\right)\tau }{3}\right)\text{FrC}\;\left(2\sqrt{\frac{D\tau }{\pi }}\right)-\cos\left(\frac{2\left(D+3J\right)\tau }{3}\right)\text{FrS}\;\;\left(2\sqrt{\frac{D\tau }{\pi }}\right)\right]$$ (5)

with the sine and cosine Fresnel functions

$$\text{FrC}\left(z\right)=\int_{0}^{z}\text{cos}\left(\pi u^{2}/2\right)\text{d}u\;\mathrm{and}$$ (6a)

$$\text{FrS}\left(z\right)=\int_{0}^{z}\sin\left(\pi u^{2}/2\right)\text{d}u$$ (6b)

and the amplitude factor *H*. All calculated spectra were generated on the basis of the above-mentioned functions. Starting values used for the simulations are noted in the respective Tables. Prior to the sine Fourier transformation to calculate the frequency spectrum, the signal was first multiplied by a Hamming window and zero-filled to double its length. Error margins of all parameters were obtained using the Cramér–Rao lower bounds theorem (Fursman and Hore, 1999).

### Matlab Routine and Algorithm

All calculations were performed using Matlab R2019a (The MathWorks, Natick, MA, United States). Unless otherwise noted, the used values were: $T_{\text{d}}=\text{0}\text{.}35\mu \text{s}$ , H=1 , probe pulse step width = 2–10 ns. Simulated OOP ESEEM decays and corresponding frequency spectra were scaled to similar amplitudes for comparability. Reconstruction was performed using the autoregressive (AR) model described below. To do this, the order *p* was increased until the reconstructed region was identified as noisy by visual inspection, and it was verified that the residuals were uncorrelated (Neumaier and Schneider, 2001). It has to be mentioned that this criterion could not be fulfilled for certain signals with high SNR; in this case, the reconstruction was optimized manually. Fitting was performed by minimizing the sum of the squares of the offsets of simulated and modeled curves using the “lsqcurvefit” routine with the trust region reflective algorithm (options: maximum iterations: 200; termination tolerance: 10^−10^; maximum function evaluations: 10,000). The squared norm of the residuals was calculated as a performance criterion in cases where fits with different starting values were compared. Boundary conditions are given in the Results and Discussion section.

### Calculation of Mass and Spin Density Centers-of-Gravity

Mass and spin density centers-of-gravity were calculated by DFT-optimized structures including all atoms of the respective molecule and by the Mulliken spin densities from the output of the respective calculation. 1-Ethyl-lumichrome and truncated tryptophan were drawn with Avogadro (Hanwell et al., 2012) (Supplementary Figure S1) and used in DFT calculations with the Orca program package (Neese, 2012). Geometry optimizations were carried out with the BP86 functional (Perdew, 1986; Becke, 1988) in conjunction with the def2-TZVPP basis set (Weigend and Ahlrichs, 2005) using the RI approximation. EPR property calculations were performed on the obtained geometries with the B3LYP functional (Becke, 1986; Lee et al., 1988) in conjunction with the EPR-II basis set (Barone, 1996) using the RICOSX approximation.

## Results and Discussion

### Published OOP-ESEEM Distances

First, we analyzed the accuracy of previously published radical-pair distances in proteins. For this purpose, previously published distances obtained by the OOP-ESEEM method were collected in Table 1 and compared with values from other structure-determination methods. To date, OOP ESEEM has been applied to only a few protein systems. Beginning in the mid-1990s, several reports were published on light-induced radical pairs in photosystems (Zech et al., 1996; Bittl and Zech, 1997; Dzuba et al., 1997; Zech et al., 1997; Santabarbara et al., 2006; Savitsky et al., 2013). Depending on the generated radical pair, distances of ≈25, ≈28 and ≈34 Å were obtained (Table 1). In this context, also oriented membranes (Yoshii et al., 1999) and single crystals (Bittl et al., 1997) of photosystems have been investigated, which allowed the determination of the orientation of the dipolar coupling axis with respect to the crystallographic axes from the angular dependence of the observed echo modulation.

In recent years, radical pairs generated in a further family of light-active proteins were investigated by this method: the photolyases and cryptochromes. The question addressed in these studies was which redox-active amino acid acts as the final electron donor in the photoreduction of the light-excited flavin adenine dinucleotide (FAD) cofactor (Aubert et al., 2000) along a cascade of tryptophan (Trp) residues. Depending on the organism and variant, either the third (TrpC) or fourth Trp (TrpD) of a conserved chain of four Trp residues (TrpA, TrpB, TrpC, and TrpD) could be identified as electron donor (Table 1) (Nohr et al., 2017; Hochstoeger et al., 2020; Xu et al., 2021). All distances yet determined by this method were found to be in very good agreement with calculated or structure data. Hence, it is evident that the method yields very good results in different systems.

It should also be noted that the distance distributions extracted from OOP-ESEEM time traces are very narrow (see the experimental time traces in the publications listed in Table 1), which is quite different from typical distance distributions from PELDOR studies of proteins (Schiemann et al., 2021). This result is unusual on first inspection because (nitroxide) spin labels, as commonly used in PELDOR spectroscopy, are rather small molecules with localized electron spin density, whereas the radicals involved in the OOP-ESEEM publications are often larger and thus have a more extended spin density distribution. The reason could be that all involved amino acids and cofactor radicals are embedded inside a larger protein (complex) and therefore have well-defined positions and orientations. In addition, their motional degrees of freedom are rather restricted. On the other hand, site-directed spin labeling of a protein is often restricted to its surface, where the linking cysteine residues are more readily accessible. However, spin centers at protein surfaces have typically larger conformational degrees of freedom, thus yielding broader distance distributions. An additional reason for the observed narrow distance distributions of OOP-ESEEM is that in the protein systems studied so far, electron transfer after light irradiation occurs stepwise *via* a chain of redox-active molecules, but the intermediate radical pairs formed in this process have a lifetime too short to contribute to the OOP signal. Thus, only the terminal, longest-lived radical pair is detected and analyzed.

### Distance Dependence of *D* and *J*

**FIGURE 2:**
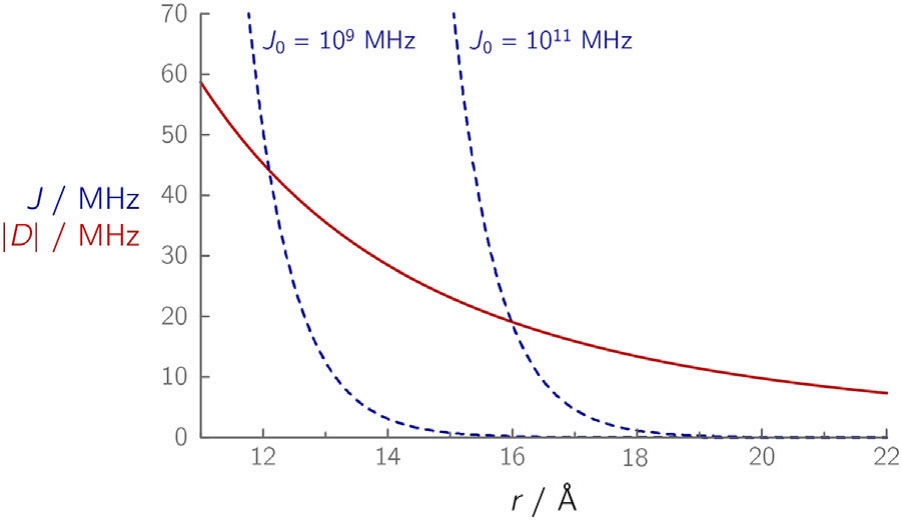
Calculated distance dependences of |*D*(*r*)| (red curve) and *J*(*r*) (dashed blue curves) in a radical pair. The spin–spin couplings *D* and *J* were calculated using Eqs. 3 and 7, respectively, using *β* = 1.4 Å^–1^, and the *J*_0_ values indicated next to the respective curves.

In a coupled radical pair, both exchange and dipolar interactions depend on the distance *r* (Figure 2): While the dipolar interaction follows an *r* ^−3^ dependence, the exchange interaction falls off more strongly with increasing distance r , following an exponential relationship: J(r)∼exp(βr) . The magnitude of D(r) can be easily estimated using Eq. 3. This is not so straightforward for J as the strength of the exchange coupling does not only depend on the distance but also on the electronic structure of the radical pair partners and the medium in between. The latter is expressed by the factor β , which parametrizes the exponential decay of the exchange coupling with the distance for a given medium (De Kanter et al., 1977):

$$J\left(r\right)=J_{0}\exp\left(\beta r\right)$$ (7)

An analogous relationship applies to the electronic coupling *V* ^2^, which is proportional to the exchange coupling and also plays a role in Marcus’ theory (Marcus and Sutin, 1985). For the electronic coupling, a value of *β* = 1.4 Å^–1^ was found for proteins (Moser et al., 1992). The magnitude of *J*_0_ is per se unknown; however, it can be estimated using Eq. 7 for systems in which *J* has been determined experimentally (Table 1). For the FAD–TrpC radical pairs (*J* ≈ 0.05 MHz) in the photolyase/cryptochrome protein family, *J*_0_ values of ≈1·10^9^ MHz were determined, while for the FAD–TrpD radical pair (*J* ≈ 0.03 MHz), *J*_0_ values are larger (≈1·10^11^ MHz) (Nohr et al., 2017; Hochstoeger et al., 2020; Xu et al., 2021). Although the radical pair composition is identical in both cases, the different *J*_0_ values (Figure 2) indicate that other parameters, in this case, differences in the protein environment, have an influence that should not be ignored. Furthermore, at larger distances, it becomes increasingly difficult to determine *J* accurately: The small *J* value in case of the FAD–TrpD radical pair in cryptochromes contains a larger uncertainty (Hochstoeger et al., 2020), which could alternatively explain the significant discrepancies between the abovementioned *J*_0_ values. As a comparison, a much larger *J*_0_ value of ≈1·10^13^ MHz was estimated for the primary radical pair of a photosynthetic reaction center (Efimova and Hore, 2008), thus demonstrating that *J*_0_ can differ by several orders of magnitude, depending on the radical pair composition and the environment in which it is embedded.

*D* and *J* are of similar magnitude in a distance range of ≈13–16 Å, Figure 2 (O’Dea et al., 2005). At distances larger than ≈17 Å, *D* becomes the dominant parameter that essentially reflects the modulation frequency of an OOP-ESEEM time trace. In all yet published studies of weakly coupled radical pairs in proteins, distances between ≈17 Å (Nohr et al., 2017; Hochstoeger et al., 2020; Xu et al., 2021) and ≈33 Å (Zech et al., 1999; Santabarbara et al., 2005) were obtained; *D* was found to be at least one order of magnitude larger than *J*.

### Applicability of the Point-Dipole Approximation

From an OOP-ESEEM experiment, the effective distance *r* between the two electron spins is, in general, determined *via* the *D* value under the assumption of the validity of the point-dipole approximation (Eq. 3). By comparing the calculated distance with a distance extracted from a structure model or from an experimentally determined structure, it is then possible to assign the involved amino acids that take place in radical pair formation. It is thus important that the applied approximation describes the structure of the molecule as accurately as possible, in particular, if two (or even more) amino acids with similar distances could potentially be a part of the coupled radical pair. Often, *r* is determined by measuring the distance between the respective atom of the highest electron spin density (“point-dipole model”) in the two radicals. For instance, in an [FAD^•–^···Trp^•+^] radical pair, these are the center between N(5) and C(4a) of the FAD^•–^ and the C(3) atom of the Trp^•+^ (Nohr et al., 2017); in a protein with two nitroxyl radicals, this would be the center of each of the two NO bonds. Alternatively, the center of gravity of the electron spin density distributions of the respective radicals can be determined either experimentally by hyperfine spectroscopy (Harmer, 2016) or theoretically, for example, *via* calculations at the DFT level of theory, and the distance between the two centers of gravities can then be determined (“center-of-gravity model”).

These two models were compared with a refined “distributed point–dipole model” (Bertrand et al., 1996), which takes the local dipoles of all pairs of atoms into account, and furthermore, with a quantum mechanical solution (Riplinger et al., 2009). Aromatic and non-aromatic nitroxides connected *via* different linker groups served as model systems; the distances between such biradical systems were determined *via* X-ray crystallography. Short distances and strongly delocalized electron spins lead to a failure of all point-dipole approximation models as *J*, and the quantum mechanical exchange part of *D* then play major roles. *J* depends on the orientation of the two radical moieties with respect to each other, as this strongly influences the overlap of the respective SOMOs. While the point-dipole approximation fails for unsaturated linkers between the two molecules even at larger distances, such models become more accurate with increasing distances for saturated linkers; in particular, the center-of-gravity approximation leads to very good results.

In principle, the distance between two coupled radicals also depends on their mutual orientation. Different orientations may result in an incorrect assignment, especially if no structure information on a system is available. The reason is that OOP-ESEEM measures the distance between the centers of the respective electron spin density distributions, which may not coincide with the centers of gravities of the two radical-pair halves. How large this error could be can only be estimated, as the uncertainty depends on the differences in the centers of gravity with respect to their spin density distributions. As an example, a [Flavin^•–^···Trp^•+^] radical pair with a distance of 20 Å was studied. Both radicals are unsymmetric, and hence, the respective centers of gravity and centers of spin density distribution do not coincide (both molecules and the corresponding electron spin density distributions are shown in Supplementary Figure S1). The molecules were rotated in 90°-steps with respect to each other without altering the distance of their centers of gravity. Depending on their respective orientation, the spin density distance varied by up to ±0.5 Å, that is, by up to 5%. This result shows that although there are only small differences between the center of mass and the center of spin density in both molecules, the influence of the orientation on the accuracy of the determined distance should not be neglected. This is especially relevant for molecules with very asymmetric spin density distributions.

### The Influence of Dipolar and Exchange Interaction on the OOP-ESEEM Time Trace

To determine the accuracy of the OOP-ESEEM method on spin-correlated radical pairs in proteins, first, the parameter space of *D* and *J* was probed (in the subsequent Figures, time traces are always depicted on the left-hand side and the corresponding spectra obtained by SFT on the right). Calculations with altered *D* parameters (−2, −6, −10, and −14 MHz) and a fixed small *J* value of +0.01 MHz were performed (Supplementary Figure S2). As expected, an increase in the absolute *D* value leads to an increase in the modulation frequency. The corresponding frequencies $\nu_{∥}$ and $\nu_{⊥}$ in the SFT spectra are clearly separated from each other and can be easily read out. Two different relaxation times *T*_d_, 0.35 and 0.1 μs, were used in each case. The corresponding SFT spectra reveal that fast relaxation only causes difficulties if it is much shorter than one oscillation period of the time traces, that is, if $T_{\text{d}}<2/\omega_{\text{ef}\text{f}}$ . This is the case only for very small absolute *D* values ( |D|<2MHz ), which correspond to large distances (>33 Å). Here the relaxation time is so short that the first maximum is shifted relative to the signal with the more inefficient relaxation. A fit and, in particular, an SFT-based analysis would lead to an incorrect result, as demonstrated by the altered frequency *ν*_∥_. Such an effect can be counteracted if a realistic *T*_d_ value can be estimated (based on data from other methods) and used as a fitting parameter. The situation improves considerably for larger absolute *D* values: As long as at least one full oscillation period can be analyzed, inflection points appear at identical positions in the SFT spectra but are less pronounced at shorter relaxation times due to the smaller number of detected modulations. This could, in principle, lead to larger uncertainties in experimental data with low SNRs.

The influence of the parameter *J* at a constant *D* value of −8 MHz is shown in Supplementary Figure S3. An increase of *J* from 0.01 to 0.1 MHz has hardly any effect on the time trace and on the frequency spectra. *J* values that are in the order of the magnitude of |D| (in this case: *J* = 0.5 and 1 MHz), however, show a clear decrease in the modulation frequency, and an increasingly strong initial rise of the signal can be detected. The reason for the frequency decrease can be explained by the different signs of *J* and *D,* as the two interactions cancel each other out to some extent (Efimova and Hore, 2008).

### Constraints in Experimental OOP-ESEEM Time Traces and Corresponding Spectra

A number of difficulties can complicate a straightforward spectral analysis of experimental data sets: 1) Observation of only a few modulations due to fast relaxation and/or a weak spin-echo signal can obscure a proper analysis. 2) ESEEM signals caused by nuclear spins are often superimposed, thus leading to additional frequencies in the OOP time trace. Fortunately, they typically have a much smaller amplitude than the dipolar modulations. If such additional frequencies preclude an unambiguous analysis, increasing the magnetic field and correspondingly the microwave detection frequency can be a simple solution (Savitsky et al., 2013; Nohr et al., 2017) as modulation depths of nuclear ESEEM frequencies depend on the magnetic field (van Doorslaer, 2017). 3) The adjustment of the proper microwave-detection phase is often rather difficult. This is due to the fact that in the vast majority of cases, no dark-stable EPR signal can be detected due to the lack of any paramagnetic species prior to sample irradiation. Improper phasing, however, may lead to nuclear ESEEM frequencies “leaking” from the in-phase channel into the OOP channel. This effect has been extensively studied previously (Fursman and Hore, 1999): Nuclear frequencies become visible in the SFT spectrum. These can, to some extent, “wash out” the outer regions of the SFT spectrum, thus making the read-out of *ν*_∥_ difficult. However, it has been shown that the superposition of nuclear frequencies does not lead to large errors in the determination of *D* (Fursman and Hore, 1999). 4) The typical deadtime of a pulsed EPR spectrometer (Stoll, 2017) precludes detection of the early response to the application of the OOP ESEEM pulse sequence (typically ≈100 ns after the last microwave pulse). Hence, one has to cope with a “truncated” time trace. In the following sections, the influence of the missing data due to the spectrometer deadtime and the reconstruction of these on the frequency spectrum obtained by SFT will be investigated.

### Reconstruction of the Early Time Points of the Time Trace

**FIGURE 3:**
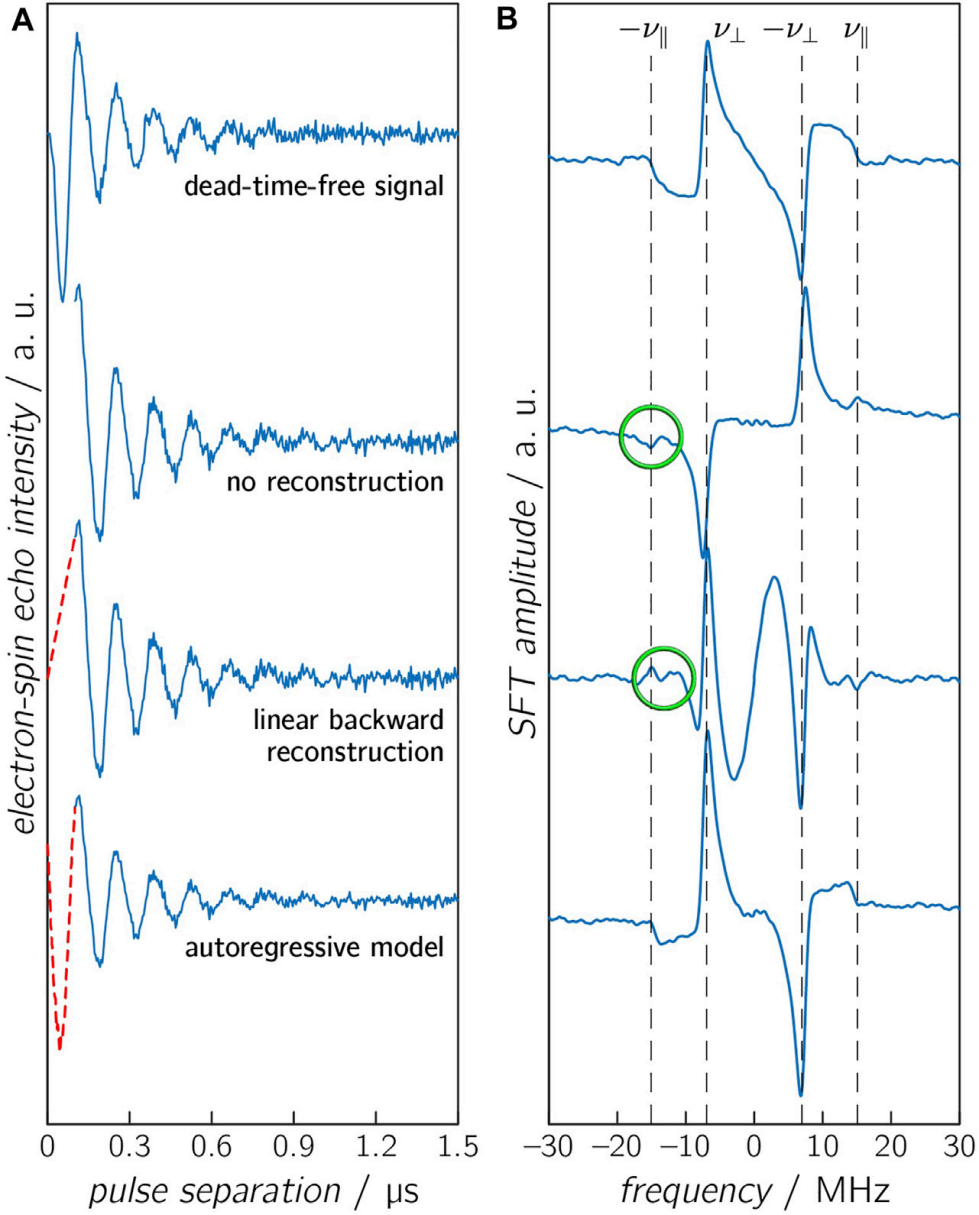
Calculated OOP-ESEEM time traces **(A)**, including noise (SNR = 50) with a spectrometer deadtime of 100 ns, and the corresponding SFT spectra **(B)**. The missing first 100 ns were either not reconstructed (second panel), reconstructed with a linear model (third panel), or reconstructed with an AR model (lowest panel). The untruncated time trace is depicted in the upper panel. The vertical dashed lines in the SFT spectra depict the frequencies *ν*_⊥_ and *ν*_∥_. Differences between calculated and reconstructed values of *ν*_∥_ are highlighted in green.

Truncation of the early part of the OOP time trace can have a certain impact on the resulting SFT spectrum. Fortunately, information on *D* and *J* is still present in the echo modulation of the later parts of the time trace, but the first part should be determined by reconstruction for a proper analysis. The influence of the spectrometer deadtime and the reconstruction of the early time trace on the SFT spectrum will be analyzed in more detail below (Figure 3). For a reasonable reconstruction, it is helpful to have a rough estimate of the exchange interaction *J*, as larger values of this parameter define the initial rise of the time trace. If *J* is much smaller than |D| , the shape of the signal is analogous to that depicted in Supplementary Figure S2. If *J* is expected to be in the range of |*D*|, initial shapes, as exemplified in the upper panel of Supplementary Figure S3, can be assumed. The OOP-ESEEM time trace can now be reconstructed using a number of different methods: 1) A linear extrapolation can be used by plotting a straight line from the first measured point to the time point at *t* = 0, which per definition has zero intensity. This method can be extended by using a polynomial extrapolation. 2) A least-squares fitting of the truncated time trace can be performed. Here, Eq. 5, which contains the parameters *H*, *T*_d_, *D*, and *J*, is fitted to the measured time trace, and the resulting optimal fitting function is then extrapolated back to the point at *t* = 0. 3) Some studies have used the “maximum entropy method” to reconstruct the missing time points (Bittl and Zech, 1997). 4) Precise predictions can be obtained using an autoregressive (AR) model (Neumaier and Schneider, 2001). Here, an *m*-dimensional AR model of order *p* is applied, which is composed of vectors *v*_*ν*_ measured at discrete time points *ν* according to the following equation:

$$\nu_{v}=w+\sum_{l=1}^{p}A_{l}\nu_{\nu -l}+\varepsilon_{\nu }$$ (8)

*A*_1_ to *A*_*p*_ are the coefficient matrices, $\varepsilon_{v}=\text{n}\text{o}\text{i}\text{s}\text{e}\left(\text{C}\right)$ are uncorrelated *m*-dimensional vectors with a covariance matrix *C* that has a mean of zero, and w is a vector of *y*-axis terms that is set to zero because the mean of the time signal should also be zero. The parameters ( A,C,w ) are assessed with a stepwise least-square fit to the (here one-dimensional) discrete time trace, with *p* as the optimization criterion (Neumaier and Schneider, 2001). The reconstruction of the data point S(k) within the experimental deadtime then takes place backwards *via* the last *k*—*l* points according to $S\left(k\right)=\sum_{l=1}^{p}A_{l}\left(l\right)S\left(k-l\right)$ until the point at *t* = 0 is reached.

Figure 3 displays time traces with identical *D* and *J* values (*D* = −11 MHz and *J* = 2 MHz), including Gaussian noise (SNR = 50), which were reconstructed by different reconstruction methods. The complete time trace (without an experimental deadtime) is shown for comparison. Additional signals appear in the SFT spectra for the time traces without reconstruction and with linear reconstruction. Linear reconstruction can additionally lead to an underestimation of the reconstructed oscillation amplitude and can cause additional modulations in the SFT spectrum due to the discontinuity of the time trace. On the other hand, the time trace reconstructed by the AR model and its respective SFT spectrum shows only minor differences compared to the deadtime-free spectrum.

It is worth mentioning that in all scenarios described above, the frequency $\nu_{⊥}$ remains virtually unchanged. On the other hand, the frequency $\nu_{∥}$ can only be read out accurately if the signal was reconstructed by the AR method, so the latter is the only one of the methods presented here that is suitable to determine the inflection point and thus $\nu_{∥}$ , which is crucial for an exact determination of both *D* and *J* parameters. A linear reconstruction is only justified if the earliest minimum or maximum can still be detected, that is, the signal falls or rises monotonically from the zero point to the first detected point. This is the case either if the deadtime is short enough or the oscillation period is long enough [see the reconstructed signals in (Dzuba et al., 1995)].

For a best-possible reconstruction, it is beneficial to have a rough estimate of how the signal behaves within the experimental deadtime. If structure data on the radical pair of interest is available, *D* can be determined *via* the point-dipole approximation, and *J* at least indirectly either by using the distance dependence shown in Figure 2 or *via* electronic coupling within the framework of Marcus’ theory (Marcus and Sutin, 1985). These estimates can then be used as initial values of *D* and *J*.

### Calculated Examples of OOP-ESEEM Time Traces

**FIGURE 4:**
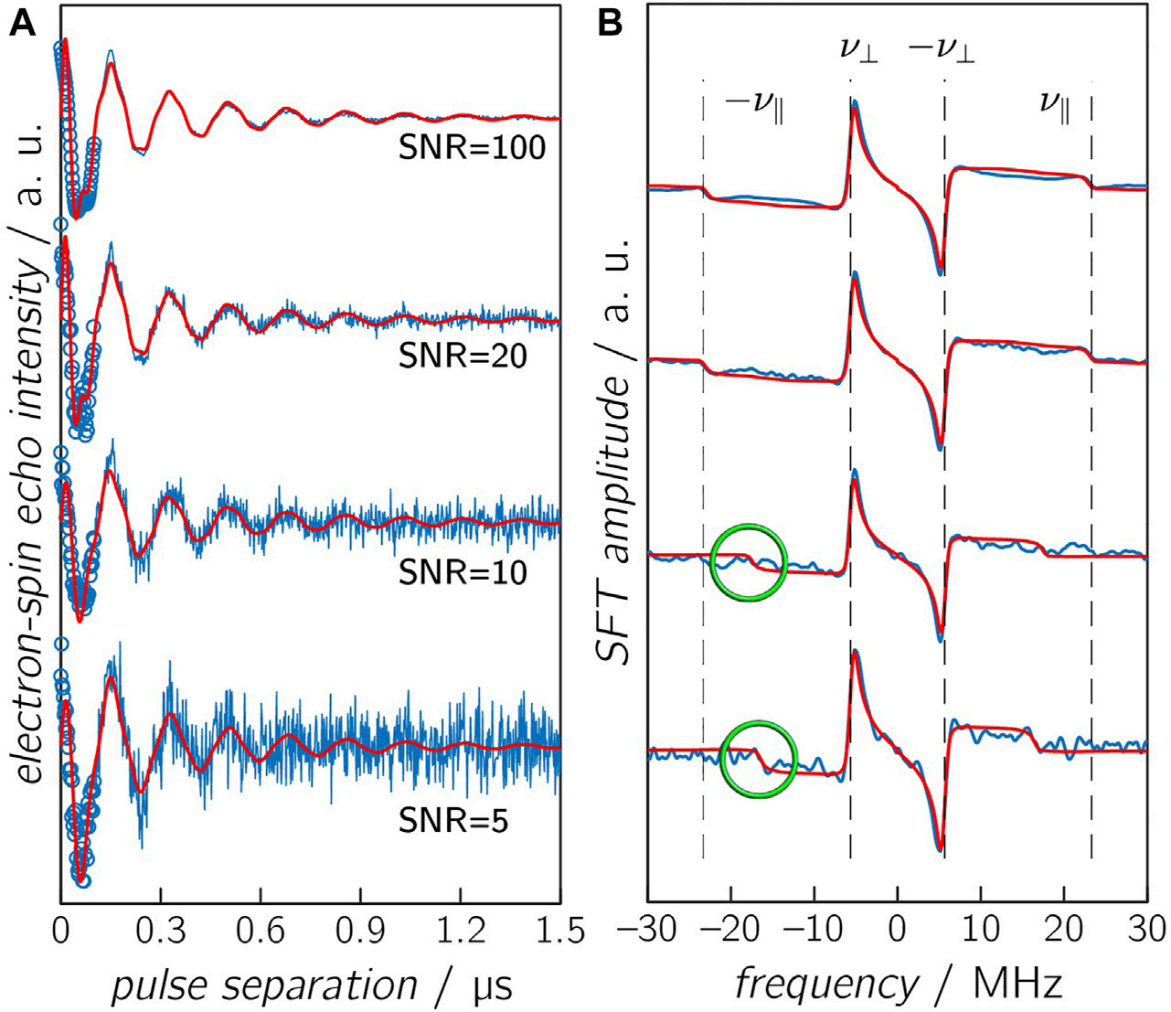
Calculated OOP-ESEEM time traces **(A)** and corresponding SFT spectra **(B)**. Calculated time trace (*D* = −14.5 MHz and *J* = 2 MHz), including reconstruction with the AR model (circles) and various SNR levels, are depicted in blue, and results from numerical simulations are depicted in red. The vertical dashed lines in the SFT spectra are the correct frequencies *ν*_∥_ and *ν*_⊥_. Please note that calculated and simulated frequency *ν*_∥_ are only identical in case of an SNR ≥20, and differences are highlighted in green. Other parameters are summarized in Table 3.

To evaluate the accuracy of the distance determination at various distances, a series of time traces using different combinations of *D* and *J* values were calculated and compared with the results of numerical simulations (Figure 4 and Supplementary Figure S4). To the calculated time traces, Gaussian noise of varying intensity was added to ensure that these data sets resemble those typically obtained from experiments. With an SNR of 100, the time trace differs only slightly from a noise-free data set. However, an SNR of 5 renders a time trace in which the modulation is only barely visible. Additional SNR values of 10 and 20 were chosen, which are in the range of typical experimental SNRs. The first 100 ns of each time trace were first truncated to mimic the spectrometer deadtime and then reconstructed using the AR model. The chosen *D* and *J* value pairs provide a good estimation of different distance ranges. At the short end, around 15 Å, *J* and |*D*| assume comparable values. Results based on the point-dipole approximation become invalid, thus making the distance determination less accurate. At the other end, at distances larger than 45 Å, the dipolar coupling becomes very small so that modulations can only be detected in case of very long relaxation times, which was reflected by using two *T*_d_ values, 0.35 and 0.10 µs. To account for the entire distance range under investigation, distances of 16.5, 17.6, 19.2, 21.4, 34, and 43.7 Å, respectively, were used, which correspond to *D* values of −17.5, −4.5, −1, −8, −2, and −1 MHz. The respective *J* values were estimated using Eq. 7 with *J*_0_ = 1⋅10^11^ MHz, resulting in values of 10.0, 2.0, 0.2, 0.01, 0.00, and 0.00 MHz.

After deadtime reconstruction, the time traces were fitted by numerical simulation to obtain optimized values for *D*, *J,* and *T*_d_ using starting values that were ±15% off the correct values. The values of *D* and *J* were obtained by the fit, and the respective SFT spectrum was calculated from the “experimental” data and from the optimal fit results. The calculated “correct” frequencies $\nu_{⊥}$ and $\nu_{∥}$ are labeled by vertical dashed lines in the respective Figures in order to judge the accuracy of the SFT spectra of the reconstituted and fitted time-domain signals. The distance between the two radicals was then determined using the fitted *D* values and Eq. 3. The corresponding results are summarized in Table 2 (for longer distances) and Table 3 (for shorter distances); exemplary time traces and corresponding SFT spectra are shown in Figure 4 for the combination *D* = −14.5 MHz and *J* = 2 MHz and in Supplementary Figure S4 (for the combination *D* = −2.0 MHz and *J* = 0 MHz).

**TABLE 2:** Comparisons of calculated OOP-ESEEM time traces using different *D* and *J* value combinations with results from numerical simulations using Eq. 5. Starting values for least-squares fittings were *T*_d_ = 0.25 µs and *H* = 1. *D*, *J*, *T*_d_, and *H* were allowed by the fitting routine to be varied in the intervals –20 MHz ≤ *D* ≤ 0, 0 ≤ *J* ≤ 10 MHz, 0 ≤ *T*_d_ ≤ 10 µs, and 0 ≤ *H* < ∞. The distance *r* was calculated using Eq. 3. Obtained distances with increased error margin (>10%) or incorrect values are highlighted in orange.

Distance and *T*_d_	SNR	Simulation parameters/MHz		Starting values/MHz		Fit results/MHz		Distance
		*D*	*J*	*D*	*J*	*D*	*J*	*r*/Å
*r* = 34.0 Å *T*_d_ = 0.35 μs	100	–2.0	0.00	–3.5	0.1	–1.997(4)	<0.01	33.94(6)
	20					–2.00(2)	<0.01	33.9(3)
	10					–2.01(3)	<0.01	33.9(5)
	5					–1.96(6)	0.00(2)	34(1)
*r* = 34.0 Å *T*_d_ = 0.10 μs	100	–2.0	0.00	–3.5	0.1	–1.96(2)	<0.01	34.2(4)
	20					–1.96(5)	<0.01	34(1)
	10					–2.10(9)	<0.01	33(1)
	5					–1.3(7)	<0.01	39(22)
*r* = 42.7 Å *T*_d_ = 0.35 μs	100	–1.0	0.00	–2.5	0.1	–1.001(1)	<0.01	42.72(6)
	20					–1.001(7)	<0.01	42.7(3)
	10					–0.99(2)	<0.01	42.8(6)
	5					–1.02(3)	<0.01	42(12)
*r* = 42.7 Å *T*_d_ = 0.10 μs	100	–1.0	0.00	–2.5	0.1	–0.97(2)	<0.01	43.1(8)
	20					–0.95(9)	<0.01	44(4)
	10					–1.0(2)	<0.01	42(6)
	5					–0.2(13)	<0.01	61(229)

**TABLE 3:** Comparisons of calculated OOP-ESEEM time traces using different *D* and *J* value combinations with results from numerical simulations using Eq. 5. Starting values for least-squares fittings were *T*_d_ = 0.25 µs and *H* = 1. The starting values for *D* and *J* were 15% off the theoretical values. *D*, *J*, *T*_d,_ and *H* were allowed by the fitting routine to be varied in the intervals –20 MHz ≤ *D* ≤ 0, 0 ≤ *J* ≤ 15 MHz, 0 ≤ *T*_d_ ≤ 10 µs, and 0 ≤ *H* < ∞. The distance *r* was calculated using Eq. 3. Obtained distances with increased error margin (>10%) or incorrect values are highlighted in orange.

Distance	SNR	Simulation parameters/MHz		Starting values/MHz		Fit results/MHz		Distance from PDA
		*D*	*J*	*D*	*J*	*D*	*J*	*r*/Å
*r* = 21.4 Å	20	–8.0	0.01	–8.5	0.0	–8.00(5)	0.00(1)	21.41(5)
	10					–8.2(2)	0.00(4)	21.2(3)
	5					–8.2(6)	0.04(7)	21.2(5)
*r* = 19.2 Å	20	–11.0	0.20	–8.5	0.1	–10.5(1)	0.00(3)	19.5(2)
	10					–10.5(1)	0.00(4)	19.5(2)
	5					–10.2(3)	0.00(7)	19.6(4)
*r* = 17.6 Å	100	–14.5	2.00	–12.5	1.0	–14.41(6)	1.96(2)	17.56(7)
	20					–14.5(8)	2.00(2)	17.53(9)
	10					–11.6(1)	1.04(4)	18.8(2)
	5					–11.2(1)	0.89(5)	19.1(3)
*r* = 16.5 Å	100	–17.5	10.00	–15.5	8.0	–17.4(1)	9.98(4)	16.4(1)
	20					–17.8(1)	10.11(5)	16.4(1)
	10					–17.2(2)	9.89(7)	16.6(2)
	5					–13.9(2)	8.83(7)	17.3(3)

Several conclusions can be drawn from such numerical simulations (Tables 2, 3): 1) At the rather long distance of 34 Å, correct distances with small error margins are obtained as long as SNR ≥5 and the relaxation time is sufficiently long (≥0.35 μs) (see Table 2). This is not surprising as *J* was assumed zero. Hence, the modulation of the time trace is governed solely by *D*, and consequently, the number of fitted parameters is reduced by one. In the SFT spectrum, one frequency is sufficient for an unambiguous assignment as *ν*_⊥_ is now ± (2/3) |*D*| (see above). Consequently, even at lower SNRs, the distance can be determined precisely from *ν*_⊥_. Making relaxation more efficient (0.1 μs) affects the accuracy of the simulation results. In this case, the obtained distance is trustworthy only at an SNR ≥10. Increasing the distance to 42.7 Å in combination with a relaxation time of 0.1 μs leads to values being trustworthy only at an SNR ≥20 (Supplementary Figure S4). This clearly shows that the relaxation time is the limiting factor for the accuracy of the distance determination at longer distances. 2) For shorter distances (Table 3), the situation becomes more complicated as *J* typically assumes non-zero values, which can be of the same order as |*D*|. The time traces in Figure 4 (*D* = −14.5 MHz and *J* = 2 MHz) show that the reconstruction at *t* = 0 does not always approach exactly zero, but the AR model is capable of reconstructing even small-amplitude oscillations with high accuracy. Therefore, good fit results were obtained for time traces with smaller *J* values (≤0.2 MHz) as long as the SNR ≥5. The fitting procedure of time traces with larger *J* values renders increasingly inaccurate distance data for low SNRs, as seen from the data of the combinations SNR ≤10 and *J* = 2.0 MHz and SNR ≤5 and *J* = 10.0 MHz (Table 3 and Figure 4). 3) The threshold SNR below which the fit yields inaccurate or even wrong distance data varies for each individual experimental data set and depends, for instance, on the choice of the starting values and the algorithm used for data reconstruction. However, it becomes evident that the accuracy of the distance determination decreases as soon as *J* is of the order of |*D*|. Furthermore, reconstruction at poor SNRs can lead to additional oscillation artifacts that actually belong to the noise. This trend is confirmed in the frequency spectra (Figure 4B). While at SNRs ≥20, both frequencies *ν*_∥_ and *ν*_⊥_ can be determined correctly, incorrect values of *ν*_∥_ are extracted at lower SNRs. This finding is not unexpected for Pake patterns as the amplitude of the SFT at *ν*_⊥_ is much larger than that at *ν*_∥_ (Weil et al., 1994), which allows an unambiguous readout of only *ν*_⊥_ even at low SNRs.

To examine artifacts of fitting in more detail, OOP-ESEEM time traces (SNR = 20) with *D* = −14.5 MHz, *J* = 2 MHz, and truncated at 100 ns were calculated and then reconstructed using the AR model (see Supplementary Table S4 and Supplementary Figure S5). Three different sets of starting values of *D* and *J* were used. Depending on those, ambiguous values for *ν*_∥_ were obtained in the SFT spectra that resulted in incorrect *D* and *J* values. This result raises the question of whether and if a quantitative distinction can be made between good and bad fit results. The analysis of the residual sum of squares (RSS) shows that in this example, the lowest value indeed corresponds to the best fit (Supplementary Table S4). Unfortunately, this cannot be generalized as such validation parameters are strongly influenced by the SNR of the measurement. Nevertheless, there are some ways to avoid ambiguous fit results: 1) The SNR of the experimental time trace can be increased as much as economically possible. However, this strongly depends on the used sample and its parameters, such as photostability and quantum yield of electron transfer. 2) The number of parameters to be fitted can be reduced manually by reading out *ν*_⊥_ from the SFT spectrum; a procedure that is reliable even at lower SNRs (Figure 4, SNR = 5), and subsequently numerically fitting the SFT spectrum using this $\nu_{⊥}$ value. 3) A global fitting algorithm can be used for the simultaneous analysis of the OOP-ESEEM time trace and its SFT spectrum. Hence, the limiting factor for the accuracy of distance determination at shorter distances is typically the determination of *ν*_∥_.

## Summary and Outlook

In this study, we evaluated the accuracy and applicability of the OOP-ESEEM method in terms of published distance determinations and investigated the influence of different parameters on the analysis of experimental data in more detail. By numerical fitting of calculated model time traces, limitations of the method were derived as a function of the SNR and the distance of a spin-correlated radical pair. Some of the difficulties encountered in experiments, such as contributions from nuclear ESEEM, incorrect phase settings, or partial orientation selection due to the limited bandwidth of applied microwave pulses, which can lead to distortions of the time traces, were not included in the calculated time traces. Therefore, we attempted to take these effects into account by considering strongly different SNRs. By evaluating the distances and their respective uncertainties obtained by spectral simulation, information on the accuracy of the method at different distance regions was obtained.

At large distances, above ≈22 Å, the analysis of OOP-ESEEM time traces is rather straightforward, as in such cases, *J* approaches 0, and the modulation of the time trace is solely governed by *D*. Therefore, only the frequency *ν*_⊥_ needs to be determined, which can be achieved even at limited SNRs and after non-perfect deadtime reconstruction of the OOP-ESEEM time trace (Table 2 and Supplementary Figure S4). The only constricting parameter is the relaxation time, which should be long enough so that at least one modulation can be detected and analyzed. At shorter radical pair distances, around 20 Å, the method still yields reliable distance data even with lower SNRs, and distances can be determined with an uncertainty of less than 0.5 Å. The reason is that *J* is still orders of magnitude smaller than |*D*|, and therefore the spectrum is still dominated by the *D* parameter. In addition, the relaxation time is, in most cases, long enough with respect to the modulation period to not limit the analysis. However, the accuracy of the method decreases when *J* and |*D*] are similar in size, which could be, depending on the value of *J*_0_, the case for distances of less than ≈18 Å. Here, unambiguous values for *D* and *J* cannot be determined without further information from independent methods. This significantly increases the uncertainty of determining distances. In principle, the method can provide excellent accuracy even in this distance range, provided good start values for the numerical simulations are available. This holds for most proteins as either crystal structure data or at least a structure model is available. Moreover, only a few amino acids are intrinsic candidates for the formation of spin-correlated radical pairs, and the number of amino acids potentially involved can usually be narrowed down, leaving only a few possibilities. Thus, realistic starting values of *D* (Eq. 3) and *J*, either from Figure 2 or Eq. 7, can be estimated. Figure 4 clearly demonstrates that, with good starting values, trustworthy distance data can be obtained even at low SNRs.

Because of the title of the special “research topic,” we have limited this study to distance determinations in proteins; however, it should be made clear that the OOP-ESEEM method, as well as its accuracy and limitations described here, can be applied just as well to all other types of molecular systems as long as they form spin-correlated radical pairs. However, due to the typically less defined structure of such systems, broader distance distributions may be expected (Popov et al., 2017; Beletskaya et al., 2020). Accordingly, the method has great potential, which has not yet been fully exploited due to the few examples investigated so far. We hope that this will improve in the near future, especially if new labeling methods become available that can be applied to both the protein and to binding partners such as DNA (Olshansky et al., 2019). It may be even possible to generate new donor-acceptor molecules (Reece et al., 2007; Weng et al., 2020) or even *de novo* designed proteins (Keller et al., 2017; Liu et al., 2018; Zollitsch et al., 2018) that can be used to perform light-induced electron transfer.

Finally, it has been suggested recently that the effect of chiral-induced spin selectivity, which, among other things, enhances the anisotropy of the reaction yield of magnetic field effects, may be detected in the in-phase channel of an OOP-ESEEM signal if applied on oriented samples (Luo and Hore, 2021). If this could be confirmed experimentally, another exciting area of application could be added to the method.
