# Supplementary material for: OOP-ESEEM Spectroscopy: Accuracies of Distances of Spin-Correlated Radical Pairs in Biomolecules
Source: Front Mol Biosci. 2022 Jun 23;9:890826. doi: 10.3389/fmolb.2022.890826 (PMC9262093; doi:10.3389/fmolb.2022.890826)
Supplement: Supplementary file 1 [file DataSheet1.pdf]

Research Topic: Structure-Function Metrology of Proteins.

OOP-ESEEM Spectroscopy: Accuracies of Distances of Spin-correlated Radical Pairs in Biomolecules

Tarek Al Said, Stefan Weber, Erik Schleicher

Institute of Physical Chemistry, University of Freiburg, Albertstr. 21, 79104 Freiburg, Germany
Phone: ++49(0)761 203-6204
Email: Erik.schleicher@pc.uni-freiburg.de

Supporting Information

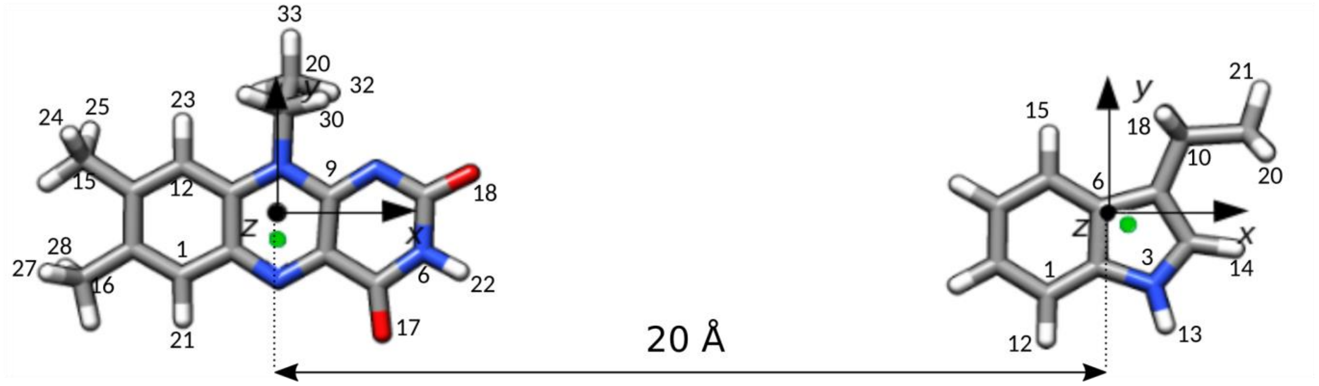

Supporting Figure 1. Comparison of center of mass (black dot) versus centers of electron spin density (green dot) of 1-ethyl-lumichrome (left) and tryptophan (right). *α*, *β*, *γ* are the angles between the *x*, *y*, and *z* axes of 1-ethyl-lumichrome and of tryptophan, respectively. The two coordinate systems are centered at the respective centers of masses. Please note that the numbering of the two molecules displayed is not in accordance with IUPAC, but as it is derived from the DFT calculations.

Supporting Table 1. Angle-dependent differences between the distance of the center of masses and the distance between the center of electron spin densities of 1-ethyl-lumichrome and tryptophan.

Orientation (*α*, *β*, *γ*) /°	Distance between the center of masses /Å	Distance between the centers of electron spin densities /Å
(0, 0, 0)	20.00	20.49
(0, 90, 0)	20.00	20.02
(0, 180, 0)	20.00	19.56
(0, 0, 90)	20.00	19.97
(0, 0, 180)	20.00	19.56
(90, 0, 0)	20.00	20.49
(180, 0, 0)	20.00	20.49

Supporting Table 2. Atomic coordinates and electron spin densities of geometry optimized 1-ethyl-lumichrome (in Å).

Atom number	Coordinates /Å			Mulliken spin densities
	x	*y*	*z*	
C1	1.22303	-0.67407	0.00723	0.164615
C2	2.47850	-0.01448	0.01839	-0.068813
N3	3.61903	-0.74756	0.01804	0.338606
C4	4.79244	-0.06420	0.01802	0.117005
C5	6.05186	-0.81114	0.00008	0.028781
N6	7.18068	0.02171	-0.00087	-0.005610
C7	7.22160	1.42819	0.01026	0.012111
N8	6.01792	2.08273	0.02254	0.009028
C9	4.89685	1.35970	0.03299	0.045522
N10	3.69485	2.08539	0.06320	0.095333
C11	2.46203	1.42287	0.02939	0.077926
C12	1.23574	2.09456	0.01099	-0.030434
C13	0.00000	1.41206	0.00000	0.174636
C14	0.00000	0.00000	0.00000	-0.064542
C15	-1.27147	2.22821	-0.00936	-0.013991
C16	-1.28429	-0.80053	-0.01058	0.004691
O17	6.18773	-2.04195	-0.01104	0.063648
O18	8.32385	1.99883	0.00561	0.039539
C19	3.75764	3.54540	0.12423	-0.003425
C20	3.64455	4.21115	-1.25088	0.005128
H21	1.25444	-1.76558	0.00440	-0.009385
H22	8.08036	-0.45235	-0.01405	-0.000287
H23	1.21288	3.18503	-0.00272	0.001271
H24	-1.36360	2.84873	0.89861	0.009773
H25	-1.30254	2.92104	-0.86713	0.008776
H26	-2.16857	1.59934	-0.06505	0.000309
H27	-1.90355	-0.60746	0.88055	-0.002492
H28	-1.90951	-0.57486	-0.88976	-0.002416
H29	-1.06245	-1.87571	-0.03106	-0.000397
H30	4.73036	3.78929	0.56657	0.000826
H31	2.96121	3.89194	0.80005	0.004282
H32	4.50431	3.92716	-1.87161	-0.000356
H33	3.64440	5.30655	-1.14428	0.000629
H34	2.72370	3.91488	-1.77147	-0.000287

Supporting Table 3. Atomic coordinates and electron spin densities of geometry optimized Tryptophan (in Å).

Atom number	Coordinates /Å			Mulliken spin densities
	x	*y*	*z*	
C1	21.20826	-0.74164	0.00040	0.077371
C2	22.38372	-0.01685	0.00107	0.010584
N3	23.71969	-0.45863	0.00186	0.153078
C4	24.55240	0.59266	0.00231	0.179348
C5	23.79511	1.80727	0.00149	0.350242
C6	22.42010	1.40309	0.00099	-0.080656
C7	21.20379	2.11609	0.00028	0.228855
C8	20.00000	1.39681	-0.00000	-0.062793
C9	20.00000	0.00000	0.00000	0.151013
C10	24.34776	3.18725	-0.00006	-0.018973
C11	25.87380	3.30011	-0.00581	0.000251
H12	21.19258	-1.83133	0.00026	-0.003468
H13	24.01476	-1.43240	0.00203	-0.007763
H14	25.63000	0.46273	0.00319	-0.009154
H15	21.19388	3.20593	-0.00011	-0.011374
H16	19.05270	1.93369	-0.00037	0.002437
H17	19.05351	-0.53946	-0.00035	-0.008009
H18	23.92763	3.72174	0.87191	0.024392
H19	23.92100	3.72156	-0.86884	0.024265
H20	26.31883	2.84094	0.88712	0.000358
H21	26.17086	4.35501	-0.01228	-0.000364
H22	26.31238	2.83150	-0.89711	0.000363

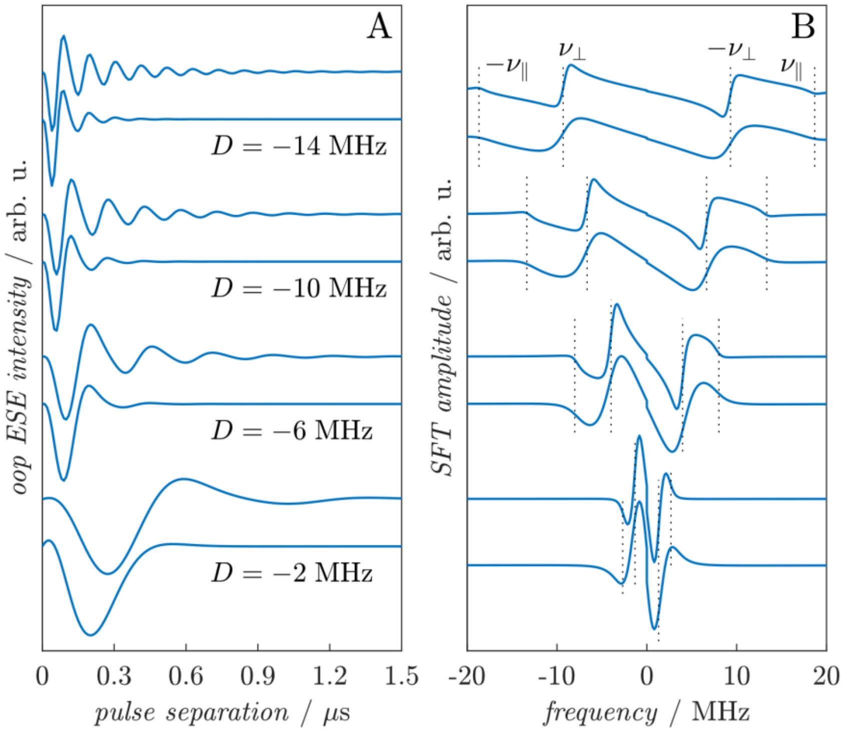

Supporting Figure 2: Influence of D values (J = 0.01 MHz) on calculated OOP-ESEEM time traces (A) and corresponding SFT spectra (B). Two different relaxation times, 0.35 μs (upper panels) and 0.10 μs (lower panels), were used. The frequencies $\nu_{\parallel}$ and $\nu_{\perp}$ are shown as dotted lines. Other parameters as in the methods section.

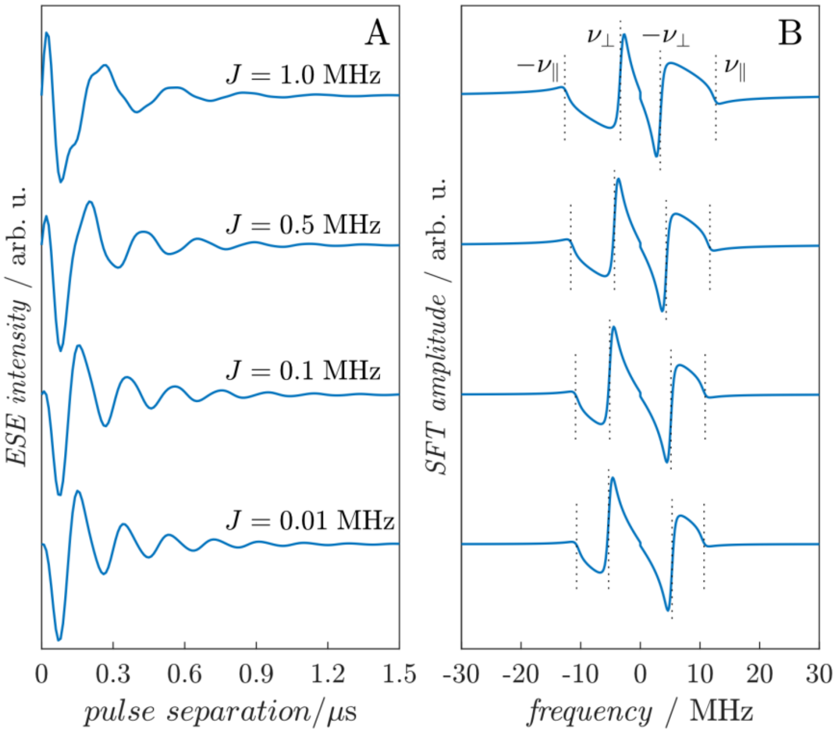

Supporting Figure 3. Influence of *J* values (*D* = –8 MHz) on calculated OOP-ESEEM time traces (A) and corresponding SFT spectra (B). The frequencies $\nu_{\parallel}$ and $\nu_{\perp}$ are shown as dotted lines. Other parameters as in the methods section.

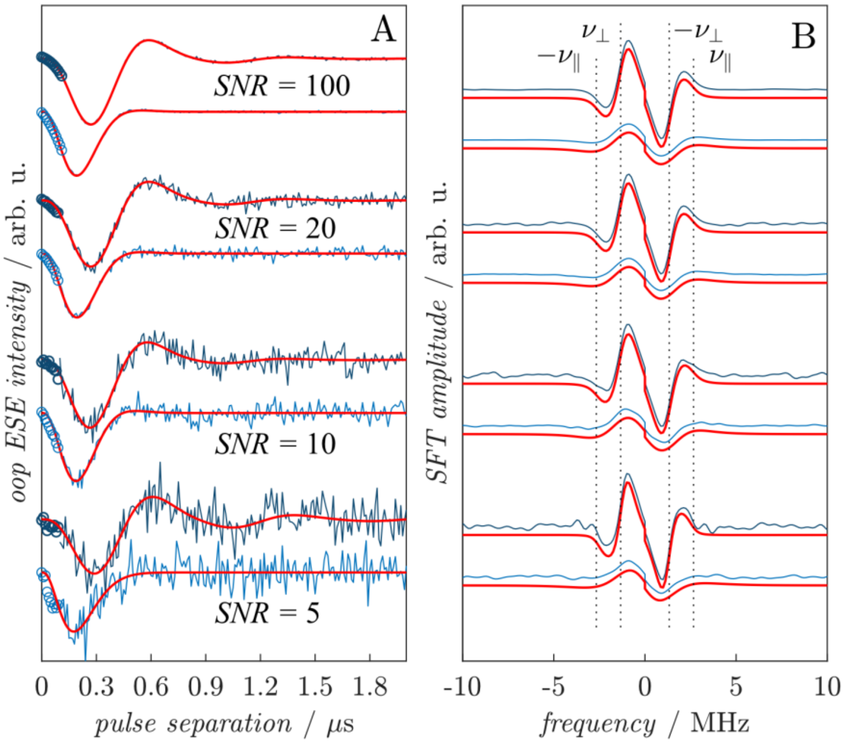

Supporting Figure 4. Influence of relaxation time and SNR on the quality of numerical simulations of calculated OOP-ESEEM time traces (A) and corresponding SFT spectra (B). Calculated time traces (D = –2.0 MHz and J = 0 MHz) with different SNRs including reconstruction with the AR model (blue circles), and two different relaxation times, T_d = 0.35 µs and T_d = 0.1 µs, are depicted in dark and light blue, respectively, results from numerical spectral simulations are depicted in red. The vertical dashed lines in the SFT spectra are the correct frequencies $\nu_\parallel$ and $\nu_\perp$. Other simulation parameters are summarized in Table 4.

Supporting Table 4. Comparison of calculated OOP-ESEEM time traces from one *D/J* pair (SNR = 20), and results of numerical simulation using different start parameters. Starting parameters for T_d and *H* were 0.25 μs and 1.0, respectively. Boundary conditions were were set to: *D* = [–20,0] MHz (fit 3: *D* = [–30,0]), *J* = [0,15] MHz, T_d = [0,10] μs and, *H* = [0,∞]. Distance *r* was calculated using the PDA. RSS: squared norm of the residual.

	D /MHz	*J* /MHz	T_d /μs	SNR		*r* /Å
Calculated parameters	−14.5	2.0	0.35	20		***r* = 17.52**
	Start parameters		Fit results			Distance from Eq. 3
	D /MHz	*J* /MHz	*D* /MHz	*J* /MHz	RSS	*r* /Å
Fit 1	−12.5	1.0	−14.50(8)	2.00(3)	27.2	**17.53(9)**
Fit 2	−11.5	1.0	−11.52(7)	1.00(2)	31.4	**18.9(1)**
Fit 3	−24.0	1.0	−22.2(1)	4.58(4)	45.6	**15.19(9)**

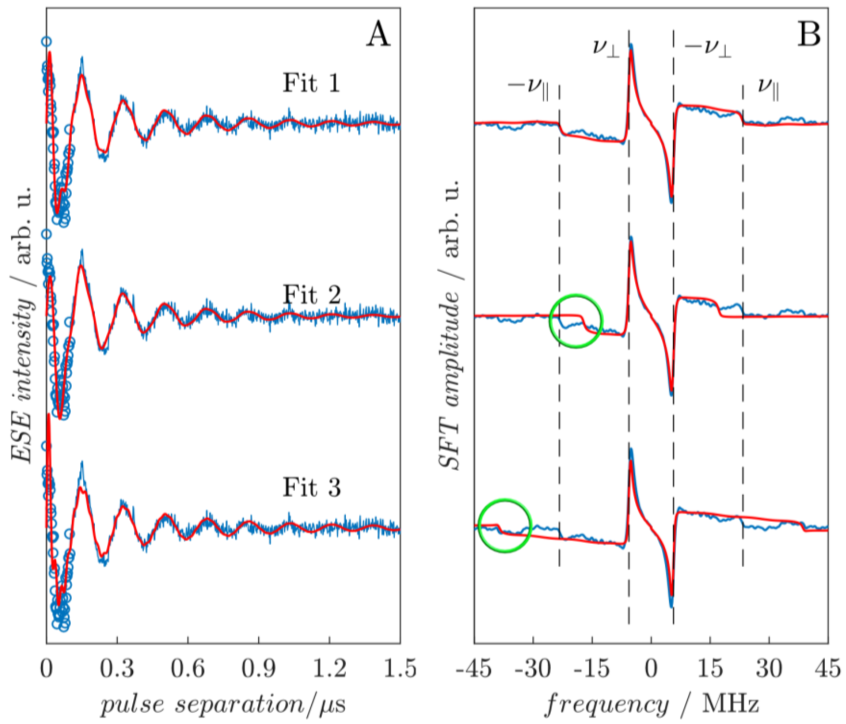

Supporting Figure 5. Influence of start parameters on the quality of numerical simulations of calculated OOP-ESEEM time traces (A) and corresponding SFT spectra (B). Calculated time traces including reconstruction with the AR model (blue circles) are depicted in blue, results from numerical spectral simulations are depicted in red. The vertical dashed lines in the SFT spectra are the correct frequencies $\nu_{\|}$ and $\nu_{\perp}$, start parameters are summarized in Supporting Table 4. Differences between calculated and simulated $\nu_{\|}$ values are highlighted in green.
